# Intra-Aortic Balloon Pump and Ischemic Cardiogenic Shock May Still Be a Valuable Association

**DOI:** 10.3390/jcm10040778

**Published:** 2021-02-16

**Authors:** Florian Rey, Raphaël Giraud, Karim Bendjelid

**Affiliations:** 1 Interventional Cardiology Unit, Division of Cardiology, Geneva University Hospitals, 1211 Geneva, Switzerland; reyflor5@gmail.com; 2 Intensive Care Division, Geneva University Hospitals, 1211 Geneva, Switzerland; Raphael.Giraud@hcuge.ch

## 1. Physiology of the Intra-Aortic Balloon Pump (IABP)

**Figure 1:**
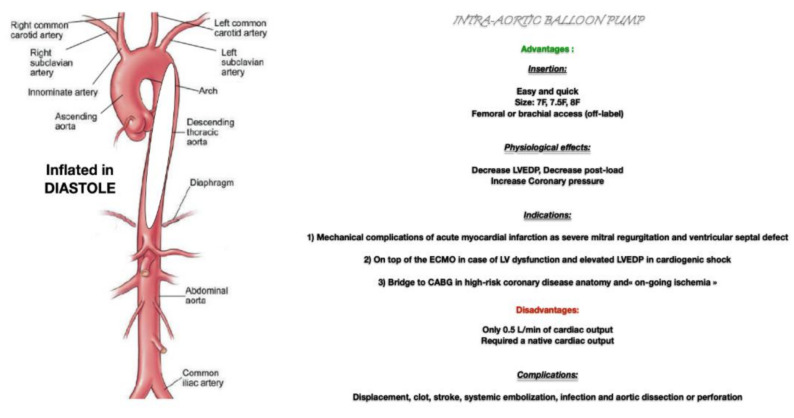
Intra-aortic balloon pump. LVEDP: Left ventricle end-diastolic pressure; ECMO: Extracorporeal membrane oxygenation; LV: Left ventricle; CABG: coronary artery bypass graft.

The IABP gives rise to greater myocardial perfusion by increasing the coronary pressure gradient from the aorta to the coronary circulation at a time when the aortic valve is closed [1]. Active deflation before the onset of systole creates a dead space in the thoracic aorta, which reduces afterload and promotes forward flow from the left ventricle. This stimulates a reduction in LV end-diastolic pressure, volume, wall tension, and work along with preservation or an increase in stroke volume and cardiac output [1]. The amplitude of the hemodynamic effect is dependent on the balloon size in proportion to the aorta and the ventricular arterial coupling. Indeed, an increase in aortic compliance and a decrease in systemic arterial tone will result in diminution of the IABP effect. Therefore, the predominant benefit of IABP on high-risk patients with severe coronary stenosis may relate to a reduction in oxygen demand through LV systolic unloading over and above that stimulated by diastolic augmentation of the coronary blood flow. Moreover, by decreasing LV end-diastolic pressure following an unloading of the LV, IABP decreases the LV wall tension and LV transmural pressure [1] (Figure 1).

## 2. Evidence-Based Medicine Concerning IABP

Few studies are available concerning the use of IABP compared to standard of care (noradrenalin, dobutamine, and intensive care unit management) or Impella mechanical support device [2,3,4] (Table 1). 

## 3. Our Point of View

As the physiopathology effect of IABP is well known, it has a crucial role to play in cardiogenic shock related to ST segment elevation myocardial infarction (STEMI), especially as most of the patients might be old with several comorbidities [5]. Indeed, patients undergoing a low cardiac output syndrome following STEMI require both an increase in systemic perfusion of all organs and a LV unloading. For instance, if an Extracorporeal membrane oxygenation (ECMO) insertion to treat a post-STEMI low cardiac output syndrome improves extra-cardiac organ perfusion, the associated increase in systemic afterload may be harmful to an ischemic heart as it decreases coronary and myocardial perfusion in a STEMI setting. In this regard, upstream insertion of an IABP from the start of ischemic cardiogenic shock seems to us a valuable option. This therapeutic method is crucial because a technique like ECMO, which is used to assist in increasing blood pressure and organ perfusion, must not impede coronary perfusion by an increase in systemic afterload, LV wall tension, and LV transmural pressure. IABP is also an inexpensive device that is easy to insert. In addition, there are different options of vascular access, such as femoral, brachial, axillary, and subclavian arteries. However, to the best of our knowledge and expert opinions, upstream insertion of an Impella in association with ECMO to unload the supported LV support in ischemic cardiogenic might also be a good option but more expensive (Figure 1) [6].

## 4. Conclusions

From our point of view, to the best of knowledge and with the lack of a pure randomized control trial comparing IABP and standard of care in ischemic cardiogenic shock, IABP in ischemic cardiogenic shock still has a role to play by itself or in association with ECMO.

## Figures and Tables

**Table 1:** Summary of the evidence.

Name	Year	Clinical Picture	Patients Number	IABP vs. Which MCS	Length of FU (Months)	Outcomes
Impress trial ^1^	2017	CS	48	Impella CP	1	No difference in mortality
IABP SHOCK II trial ^2^	2012	CA and CS	600	Control (standard of care)	12	CA: no difference in mortality;CS: no difference in mortality
ISAR-SHOCK trial ^3^	2008	CS	25	Impella 2.5	1	No difference in mortality

CA = cardiac arrest; CS = cardiogenic shock; IABP = intra-aortic balloon pump; MCS = mechanical support device; FU = follow-up;^1^ Percutaneous Mechanical Circulatory Support Versus Intra-Aortic Balloon Pump in Cardiogenic Shock after Acute Myocardial Infarction; ^2^ Intra-aortic balloon pump in acute myocardial infarction complicated by cardiogenic shock; ^3^ Efficacy Study of LV Assist Device to Treat Patients with Cardiogenic Shock.
